# Clinical Use of Proteasome Inhibitors in the Treatment of Multiple Myeloma

**DOI:** 10.3390/ph8010001

**Published:** 2014-12-24

**Authors:** Noah M. Merin, Kevin R. Kelly

**Affiliations:** Internal Medicine, Jane Anne Nohl Division of Hematology, Keck School of Medicine of University of Southern California, Los Angeles, CA 90033, USA; E-Mail: noah.merin@med.usc.edu

**Keywords:** multiple myeloma, proteasome inhibitors, toxicity

## Abstract

Multiple myeloma (MM) is an incurable hematological malignancy characterized by the clonal proliferation of neoplastic plasma cells. The use of proteasome inhibitors in the treatment of MM has led to significant improvements in outcomes. This article reviews data on the use of the two approved proteasome inhibitors (bortezomib and carlfilzomib), as well as newer agents under development. Emphasis is placed on the clinical use of proteasome inhibitors, including management of side effects and combination with other agents.

## 1. Introduction

Multiple myeloma (MM) is a malignant neoplasm characterized by clonal proliferation of plasma cells in the bone marrow that produce monoclonal protein (M-protein) that may be detected in the serum or urine. It is the second most common hematologic cancer in the United States (after Non-Hodgkin Lymphoma); an estimated 24050 new cases and 11090 deaths will occur due to MM in the USA in 2014 [1]. It is a disease of the elderly with a median age at diagnosis of 69 years in the USA [2].

Survival rates for active symptomatic MM have increased dramatically over the past 15 years. The first significant improvement was the introduction of autologous bone marrow transplantation (autoHSCT) in the 1980s. This was followed by the introduction of therapies with anti-angiogenic, anti-proliferative and immunomodulatory effects (thalidomide and derivatives; IMiDs), and proteasome inhibitors such as bortezomib and carfilzomib. Prior to the introduction of autoHSCT (during the 1970s), median overall survival for patients with MM was 24–36 months; after the introduction of autoHSCT, survival increased to 36–48 months. The introduction of thalidomide derivatives and bortezomib has increased median overall survival by up to 7–8 years [3,4].

MM is not curable with current treatments, and patients will typically receive several lines of therapy over the course of treatment. Patients are stratified based on eligibility for autoHSCT (determined by age and medical comorbidities); patients who are eligible for transplant receive induction therapy. Induction regimens vary widely, but in the USA they typically incorporate bortezomib and/or lenolidomide with an alkylating agent and steroids. Transplant-eligible patients then undergo harvesting of autologous stem cells followed by high-dose melphalan and autoHSCT. Following autoHSCT, patients may receive consolidation and/or maintenance therapy with prolonged treatment employing single-agent bortezomib or lenolidomide. Patients who are ineligible for autoHSCT receive combination therapy with bortezomib, an alkylating agent, and steroids, or bortezomib and lenolidomide with steroids, followed by maintenance therapy [5].

Proteasome inhibition has assumed a central role in the management of MM, due to the effectiveness of this treatment strategy, a manageable safety profile, and the ability to combine proteasome inhibitors with other chemotherapeutic agents. As an indication of the level of scientific interest in using proteasome inhibitors in combination with other agents, we note that there are currently over 120 clinical trials listed on ClinicalTrials.gov actively recruiting patients with MM for experimental regimens incorporating bortezomib, and many other trials using second- and third-generation proteasome inhibitors. There are 328 published articles listed on PubMed that report original clinical trial data on the use of bortezomib in MM. Bortezomib is used at every stage of treatment for MM, from frontline combination therapy, to re-treatment for relapsed disease, combination therapy for refractory disease, and as induction, conditioning, consolidation, and maintenance therapy before and after autoHSCT [6,7,8,9].

The rationale for targeting the proteasome in MM is that malignant plasma cells typically produce a large amount of immunoglobulin that eventually has to be broken down, leading to a higher level of proteasome activity than in normal cells. Therefore, MM cells undergo apoptosis more readily when protein homeostasis is disrupted [10]. This confers selectivity to these agents and a wide therapeutic index that is non-cell cycle specific (unlike cytotoxic chemotherapeutic agents, which affect all dividing cells and derive their selectivity from the fact that a larger fraction of the cancer cells are undergoing mitosis at any given time, compared to normal cells).

## 2. Structure and Function of the Proteasome

The proteasome is a barrel-shaped multi-enzyme complex, formed from four homologous rings (two alpha rings and two beta rings). The inner surface of the barrel contains six proteolytic centers that selectively cleave malformed, damaged, or unnecessary proteins using trypsin-like, chymotrypsin-like or caspase-like catalytic activity. Ubiquitin-tagged whole proteins enter through a regulatory subunit at one end of the proteasome, undergo lysis, and exit as peptide fragments at the other end [10,11,12,13].

The proteasome is not the only system responsible for handling misfolded proteins. Other mechanisms include up-regulating chaperones to assist protein refolding, autophagy–lysosome systems, and formation of aggresomes which transport the misfolded proteins along microtubules for lysosomal clearance. Of these, the proteasome is the most important system for the recognition and degradation of damaged proteins (70%–90% of the misfolded, damaged, or no longer needed proteins are degraded via the proteasomal pathway) [11]. However, in the setting of proteasome inhibition, aggresome formation may assume heightened importance for disposing of unfolded and misfolded proteins, and protein disposal via the aggresome system may represent an escape mechanism capable of conferring resistance to proteasome inhibitor therapy. Aggresome formation depends on histone deacetylase 6 (HDAC6) activity; this observation forms the rationale for the strategy of dual protein homeostasis interference with a proteasome inhibitor and an HDAC inhibitor [14].

A special inducible form of the constitutive proteasome (found in all cells), called the immunoproteasome (found mainly in lymphoid cells), is produced under stress conditions (such as bacterial or viral infection) in the presence of inflammatory cytokines such as IFN-gamma, TNF-alpha, and bacterial lipopolysaccharides. The major function of the immunoproteasome is the production of a specific short oligopeptides that can be presented by the major histocompatibility complex class I (MHC-I) on the cell’s surface during an immune response. Immunoproteasomes are hypothesized to promote the presentation of normal self-protein fragments to prevent autoimmune responses directed at uninfected lymphoid cells [11].

## 3. Putative Mechanism of Apoptosis Induced by Proteasomal Inhibition

Proteasome inhibitors target the 20S subunit of the proteasome, which is responsible for degrading ubiquitin-tagged proteins that have been marked for removal from the cytoplasm. There are several hypotheses for the mechanism of action of these agents in MM, including: accumulation of incompatible regulatory proteins within the cell [15]; accumulation of mis-folded proteins from the endoplasmic reticulum (“ER stress”) which trigger an unfolded protein response that leads to suspension of protein synthesis [15,16,17]; and inhibition of NF-**κ**β signaling pathway (critical for the coordination of cellular responses to stress/inflammation) via interference with degradation of proteins that inhibit NF-**κ**β (I**κ**β) [18]. The effectiveness of proteasome inhibition in killing MM cells may reflect a heightened importance of protein homeostasis for these malignant plasma cells, which have a large endoplasmic reticulum and manufacture immunoglobulin proteins constitutively [16]. Due to the highly proliferative nature of these cells, intracellular signaling driving cell cycle transitions from S to G to metaphase occurs at a higher rate; when protein degradation is interrupted, these incompatible signaling proteins accumulate at a faster rate than in normal cells, which renders MM cells more vulnerable to proteasome inhibition than non-neoplastic cells.

The selective MM cell-killing ability of single-agent bortezomib has been demonstrated *in vitro* in human MM cell lines [19]; in these studies, induction of apoptosis was quantified by analysis of cell cycle arrest, DNA fragmentation, and caspase 3 cleavage. Cultures of peripheral blood mononuclear cells from normal volunteers showed only minor growth inhibition or apoptosis at IC_50_ drug levels which effectively inhibited MM cells, which indicted excellent selectively of bortezomib for MM cells compared to normal cells. Similarly, *in vivo*, in a human plasmacytoma xenograft mouse model, single agent bortezomib was shown to decrease tumor growth, prolong survival, increase tumor apoptosis, and decrease blood vessel density in tumors, without causing reductions in peripheral blood cell counts [20].

One of the most striking properties of proteasome inhibitors is their remarkable synergy with other chemotherapeutic agents, and ability to restore sensitivity to chemotherapy to chemoresistant MM cells. *In vitro* experiments with chemoresistant MM cell lines have demonstrated that this synergy is on the order of 100,000–1,000,000-fold increase in sensitivity. For example, when chemoresistant MM cell lines (developed by exposing MM cells to increasing concentrations of chemotherapy, as well as MM cell cultures from bone marrow aspirates of patients with chemoresistant disease) are treated with melphalan, mitozantrone, or doxorubicin at concentrations to which the cells were previously resistant plus a non-cytotoxic dose of bortezomib (at a concentration that does not affect normal hematopoiesis), growth curves are shifted to the left and a marked increase in apoptosis is observed [21]. The mechanism of the synergistic effect of proteasome inhibition with other chemotherapy agents is an active area of research; note that proteasome inhibition itself is a relatively non-specific condition which could augment or enhance the actions of other drugs via the multiple pathways mentioned above [10]. Proteasome inhibition may enhance chemosensitivity by interfering with cellular responses to damage induced by chemotherapeutic agents (e.g., DNA repair, apoptosis, cell cycle progression). A review of the hypotheses for the mechanistic bases of synergism has recently been published [22].

## 4. Mechanisms of Bortezomib Resistance

MM remains an incurable disease, and patients who are treated with proteasome inhibitors will ultimately develop resistance and experience clinical progression. It is of great interest therefore to characterize mechanisms of proteasome inhibitor resistance so that clinical strategies can be developed to mitigate or delay the development of resistance, and to guide the selection of subsequent therapies to address resistance mechanisms.

Initial studies conducted using bortezomib-resistant cell lines (developed by exposing cells to increasing concentrations of bortezomib) identified mutations that affected the shape of the S1 pocket of the **β**5 subunit of the proteasome (which is responsible for chymotrypsin-like activity) as possible candidate mutations conferring bortezomib resistance [23,24]. However, the clinical relevance of this finding is doubtful as proteasome **β**5 subunit mutations have not been identified in any patients with MM who demonstrate resistance to proteasome inhibitor therapy.

Recent discoveries have implicated changes in cellular pathways involved in glutathione metabolism and defense from oxidative injury in bortezomib resistance. In bortezomib-resistant cell lines, inhibiting MUC1 (a component of the oxidative defense system which is aberrantly expressed in most MM cells) induces reactive-oxygen species mediated cell death and reverses bortezomib resistance. Simultaneous inhibition of MUC1 and the proteasome results in down-regulation of the p53-inducible regulator of glycolysis and apoptosis (TIGAR) that in turn results in increased oxidative damage and cell death [25,26]. This intriguing finding awaits confirmation in bortezomib-resistant MM cells obtained from patients.

Other mechanisms of resistance that have been observed in MM cell lines and disease models include up-regulation of heat-shock proteins (such as HSP90 and HSP27) which function as ubiquitin chaperones, which may facilitate NF-**κ**B signaling, increased efflux transporter P-gp expression (which lowers intracellular bortezomib concentrations), up-regulation of proteasome subunits and increased basal proteasome activity, upregulation in Il-6, IGF-1 and other cytokines in the MM microenvironment, and increased growth signaling through insulin-like growth factor (IGF) and other pathways [27,28].

Gene expression signatures associated with bortezomib sensitivity and bortezomib resistance have been characterized in cell lines derived from transgenic mouse models of MM [29]. By comparing the gene expression profile in bortezomib-resistant cell lines to gene expression profiles from other chemo-resistant cell lines in a large database (Connectivity Map Database, Broad Institute) the authors were able to predict drug class sensitivity in the bortezomib resistant cell lines (for example, HDAC sensitivity, heat-shock protein 90 inhibitor sensitivity, cell cycle inhibitor sensitivity, *etc.*). If this approach can be developed into a clinical assay, it may be possible in the future to use gene expression profiling to predict which patients who develop proteasome inhibitor resistance will respond to treatment with other proteasome inhibitors, and rationally the next treatment, or choose additional drugs to add to restore proteasome inhibitor sensitivity. For example, if the gene expression profile suggests that the patient’s MM cells will be sensitive to combined HDAC inhibition and proteasome inhibition, this combination could be used instead of empirically switching to non-proteasome inhibitor therapy.

## 5. Clinical Aspects of Specific Proteasome Inhibitors in Use or Under Development

Please refer to Table 1 for a summary of proteasome inhibitors in clinical development and noted toxicities.

**Table 1:** Proteasome inhibitors in clinical development.

Compound	Company	Route	Phase of Development	Major Reported Toxicities
Bortezomib	Millennium	I.V./SQ	Approved	PN, hematologic toxicities, diarrhea, fatigue, dyspnea, zoster reactivation
Carfilzomib	Onyx	I.V.	Approved	Hematologic toxicities, pneumonia, hyponatremia, fatigue, hypophosphatemia, infusion reactions, chest pain, heart failure
Oprozomib (ONX-0912)	Onyx	Oral	Ib/II	Not yet reported
Ixazomib (MLN9708)	Millennium	Oral/I.V.	I/II	Hematologic toxicities, fatigue, rash, decreased appetite, diarrhea, vomiting
Delanzomib (CEP-18770)	Teva	Oral	Terminated	-
Marizomib (NPI-0052)	Nereus	Oral/I.V.	I/II	Fatigue, nausea, vomiting, dizziness, headache, diarrhea, constipation, anorexia, dyspnea, cognitive changes, hallucinations

PN, peripheral neuropathy; IV, intravenous; SQ, subcutaneous.

### 5.1. Bortezomib

Bortezomib is a boronic-acid based reversible proteasome inhibitor that targets the chymotrypsin- and caspase-like moieties of the constitutive and immunoproteasome. Phase 2 studies demonstrating the effectiveness of this agent in relapsed/refractory MM were reported 11 years ago [30]. In the SUMMIT study of 202 patients with relapsed MM, 27% of patients achieved a partial response (PR) or complete response (CR), and median time-to-progression was 7 months. The United States Food and Drug Administration (FDA) approved the drug for use in relapsed/refractory MM in 2003 on the basis of this study. In 2008, the VISTA trial presented evidence demonstrating the superiority of bortezomib plus melphalan and prednisone (VMP) compared to MP alone for patients with newly-diagnosed MM, which lead to FDA approval for use of bortezomib combinations in frontline therapy [31]. In the VISTA trial, the median time-to-progression (TTP) was 24 months for VMP *versus* 16.6 months with MP. After initial treatment, many patients remain sensitive to bortezomib and can be re-treated upon relapse; 63% of patients who responded to initial treatment with bortezomib respond to retreatment, with a median TTP of 9.3 months [32].

### 5.2. Side Effects of Bortezomib

The principle dose-limiting toxic effects of bortezomib are peripheral neuropathy (PN) [33,34], thrombocytopenia, neutropenia, anemia, fatigue, and diarrhea [9]. In the Phase II SUMMIT and CREST study with bortezomib, more than 80% of patients had symptoms of polyneuropathy [35]. It is important to note that many of these patients may have had neuropathy prior to treatment with bortezomib, as this is a common complication of MM itself, as well as other drugs used to treat MM including vinca alkaloids and IMiDs. Weekly dosing (instead of twice per week) [36] and subcutaneous administration (instead of intravenous) [37,38] have been shown to reduce the incidence of neuropathy [39] without compromising effectiveness. Thrombocytopenia is observed in almost all patients treated with bortezomib; the platelet count drops during the first 14 days of treatment, then recovers to baseline around day 21 (the average reduction in platelet count is 60% for relapsed/refractory patients) [36].

### 5.3. Combination Studies

Bortezomib has been combined with many other agents, and in general, is not used as a single agent except in the maintenance setting (after autoHSCT or upon completion of upfront therapy resulting in remission). Combination regimens incorporating alkylating agents, corticosteroids, and/or IMiDs are used for all other phases of MM therapy, and excellent reviews describing these studies have been published recently [6,7,9,40,41,42]. These combinations are effective, but many patients will ultimately develop resistance to combinations of proteasome inhibitors and IMiDs, and there is therefore a need for new synergistic combinations incorporating drugs with different mechanism of action. Here, we highlight several emerging combination strategies:

Three Drug Induction regimens: The combination of bortezomib, lenalidomide, and dexamethasone (VRd) has been shown to be highly effective and well tolerated in prospective phase II studies and is currently being in phase III trials [43,44]. In the Phase II trial, PR rate with VRd was 100%, with 74% achieving very good partial response (VGPR) or better [43]. VRd is frequently used as initial therapy in the United States despite the absence of randomized data demonstrating superiority over other induction regimens. Another commonly used induction treatment is the combination of cyclophosphamide, bortezomib and dexamethasone (CyBorD) [45]. In the Phase II trial with this regimen, the response rate (≥PR) was 88%, with 61% achieving VGPR or better and 39% achieving CR or near-CR [45]. Melphalan is an alkylating agent that is highly active in MM, but is toxic to stem cells and makes harvesting for autoHSCT difficult. CyBorD uses cyclophosphamide, which is a less stem cell toxic alkylating agent, allowing adequate stem cell collection in all patients at relatively low cost. Unlike the VRd regimen, which contains lenalidomide (which is renally cleared), CyBorD can be safely used in renal failure. While formal cost estimates comparing these two three-drug regimens that include bortezomib are lacking, CyBorD is associated with lower monthly cost in the United States and is potentially less toxic than other induction regimens.

Bortezomib + Oral Histone De-Acetylase Inhibitor (HDACi): HDAC enzymes modulate the activities of many oncogenes and tumor suppressor genes at the epigenetic and protein level. HDACs alter chromatin conformation by removing acetyl groups, which prevents the transcription of genes that encode proteins involved in cell cycle control and apoptosis. In addition to histone targets, HDACs also directly acetylate cytosolic proteins involved in intracellular transport and cell motility. Among these non-histone targets are proteins involved in the aggresome system, a non-proteasome protein degradation pathway that disposes of misfolded proteins by transporting them along microtubules to lysosomes [46]. Therefore, given the importance of protein homeostasis in MM, one approach to synergistic therapy is to simultaneously inhibit both the proteasome and aggresome systems [14,46,47,48]. Pre-clinical and early clinical studies have demonstrated support for synergistic effect of bortezomib and HDACi in MM [49,50,51]. Vorinostat is an orally available HDACi approved for the use of cutaneous T-cell lymphoma. Additional preclinical studies suggest that MM patients with elevated Myc activity may be particularly sensitive to the bortezomib and vorinostat combination [52]. A phase III randomized trial of 637 patients (VANTAGE 088) compared the combination of bortezomib plus vorinostat to bortezomib plus placebo in relapsed, non-refractory MM progressing after one to three prior regimens [49]. The addition of vorinostat was associated with a higher ORR of 56 *versus* 41 percent, and a small but statistically significant increase in the median progression-free survival: 7.73 *versus* 7.03 months. More grade 3–4 thrombocytopenia, fatigue, nausea, and diarrhea were noted with the combination regimen than with bortezomib alone. Given the minimal overall benefit and increased toxicities, future trials may incorporate revised dosing schedules or the use of newer, more specific HDACi (e.g., HDAC6 inhibitors, which may confer more specific inhibition of aggresome formation and minimize off-target effects). Panobinostat is an oral pan-HDACi that potently inhibits HDAC enzymes and decreases aggresome formation in preclinical models [53]. PANORAMA 2, a recent Phase II clinical trial, tested panobinostat in combination with bortezomib and dexamethasone in 55 patients with relapsed and bortezomib-refractory MM. The ORR was 34.5% (1 near-CR and 18 PR), which is significant in this difficult to treat population. Median exposure and progression-free survival were 4.6 and 5.4 months, respectively. In patients who achieved a response, median time to response was 1.4 months, and median duration of response was 6 months [54]. This combination is being studied in a large phase 3 trial (PANORAMA 1) in relapsed and relapsed and refractory patients with MM.

Bortezomib + Oncolytic Virotherapy (Reovirus Therapy): Reoviruses are ubiquitous virus found in human respiratory and GI flora. Reoviruses selectively replicate in transformed cells and not in normal tissue (this phenomenon is thought to be due to abnormal Ras pathway activation in cancer cells, which inhibits the activation of the normal anti-viral responses to double-stranded RNA). A reovirus-based therapy (Reolysin) is currently being tested in clinical trials in several types of cancer; preclinical data suggests that reovirus therapy may exert synergistic effects with proteasome inhibition via viral augmentation of endoplasmic reticulum stress (ER stress) [55]. Reovirus replication in MM cells delivers a large quantity of viral proteins to the ER for folding and post-translational modification; these viral proteins overwhelm the capacity of the ER and trigger ER-stress mediated apoptosis. Since proteasome inhibition also causes the accumulation of misfolded proteins which stress the ER and trigger apoptosis, Kelly *et al.* tested for Reolysis/bortezomib synergy in MM cell lines and a mouse MM xenograft model. In MM cell lines, Reolysin-mediated apoptosis was associated with an induction of ER stress-related gene expression, swelling of the ER, increases in intracellular calcium levels and a strong induction of the Bcl-2 homology 3 (BH3)-only pro-apoptotic protein NOXA [56]. Co-administration of Reolysin and bortezomib resulted in the dual accumulation of viral and ubiquitinated proteins, which led to enhanced ER stress, NOXA induction and apoptosis [56]. The combination of reovirus infection and proteasomal inhibition significantly decreased tumor burden in a xenograft and syngeneic bone disease model of MM without exhibiting adverse side effect [56]. A phase I study of Reolysin in relapsed/refractory MM has been completed [57]. The agent has been shown to be safe and minor responses and prolonged disease stabilization was observed. Combination studies of Reolysin with both bortezomib and carlfilzomib are planned.

### 5.4. Second-Generation Proteasome Inhibitors

Carfilzomib: Boronic acid containing proteasome inhibitors such as bortezomib inhibit both the chymotrypsin-like and the caspase-like activities of the proteasome. Carfilzomib is a second-generation proteasome inhibitor that selectively inhibits the chymotrypsin-like activity of the proteasome and is active in bortezomib-resistant patients. Another major difference is reversibility: whereas bortezomib demonstrates reversible effects (duration of proteasome inhibition lasts about 72 h), carfilzomib induces irreversible inhibition (once carfilzomib binds to its active site within the barrel of the proteasome, the proteasome is permanently inactivated and new proteasomes must be synthesized to restore proteasome activity). Carfilzomib was approved in 2012 for the treatment of patients with MM who have received at least two prior therapies including bortezomib and an IMiD, and have demonstrated disease progression on or within 60 days of completion of the last therapy [58]. As a monotherapy in phase II studies, carfilzomib induced an ORR of 20% in patients refractory to bortezomib [59,60,61]. Carfilzomib appears to be less likely to cause PN [62], and is safe in patients with renal impairment [63]. Phase III studies of carfilzomib are ongoing. Higher responses are observed when carfilzomib is used in combination with other agents such as lenalidomide, and low-dose dexamethasone [64,65]. The current FDA-approved dose of carfilzomib is 20 mg/m^2^ for cycle 1, and 27 mg/m^2^ for cycle 2 (20/27). Recent data have shown that the maximum tolerated dose (MTD) of carfilzomib is higher (20 mg/m^2^ for cycle 1, 56 mg/m^2^ for cycle 2 (20/56) [66]. Therefore a phase II study is underway to compare the two different doses of carfilzomib in combination with dexamethasone in a randomized fashion to determine if a higher dose can improve the efficacy while maintaining a safe toxicity profile.

Oprozomib (ONX-0912): Oprozomib is a structural analog of carfilzomib that is orally bioavailable [67]. Oprozomib demonstrated clinical activity in a phase 1 trial in patients with hematologic malignancies (MM and chronic lymphocytic leukemia) [68]. A once-daily administered tablet was introduced in a phase 1b/2 trial in order to improve gastrointestinal tolerability, and is demonstrating a good safety profile and promising preliminary response data [69].

Ixazomib (MLN9708): Ixazomib is a boronic-acid containing peptide with chymotrypsin- and caspase-like proteasome inhibitory activity, formulated for oral administration. Clinical trials are underway to assess its effects in bortezomib-refractory patients. Early indications suggest that ixazomib may be associated with less PN than bortezomib [70].

Delanzomib (CEP-18770): Delanzomib is a boronic-acid containing peptide formulated for oral administration, with a more favorable toxicity profile than bortezomib (no neurotoxicity, but noted to cause rash). Phase II trial of this drug has been terminated due to lack of efficacy in relapsed/refractory MM (delanzomib is very similar to bortezomib and is unlikely to overcome bortezomib resistance) [71].

Marizomib (NPI-0052): Marizomib is a novel non-peptidic, orally active, irreversibly-binding proteasome inhibitor with broad activity at all three catalytic sites of the proteasome [13]. Since it is not peptide-based, marizomib is resistant to degradation by endogenous proteases. It is capable of overcoming bortezomib resistance *in vitro*; clinical trials are underway but are still early stage. Dose-limiting toxicities in phase I trials have included cognitive changes, transient hallucinations, and loss of balance, which were reversible. The most common drug-related adverse effects included fatigue, gastrointestinal AEs, dizziness, and headache. There was no evidence of PN or thrombocytopenia [71]. Since marizomib has a different mechanism of action from bortezomib, and a non-overlapping toxicity profile, combinations of these agents may be evaluated in future studies [71].

This is a very active area of research; there are over 10 structurally-distinct classes of proteasome inhibitors in development, with new agents expected to enter clinical testing in the hopes of finding drugs with optimal potency, reduced toxicity and oral bioavailability [72]. Table 2 outlines some of the ongoing clinical trials investigating the combination of second-generation proteasome inhibitors with other agents.

## 6. Toxicities of Proteasome Inhibitors

Given their broad application and use in combination with other agents with similar associated toxicities (especially vinca alkaloids and IMiDs), potential for patients to be exposed to years of proteasome inhibitor therapy, and the fact that patients can live for years with side effects when treatments are successful, there has been a considerable focus on characterizing and mitigating the side effects of proteasome inhibitors since their inception. One of the main impetuses for the development of the second-generation agents has been to reduce the occurrence of PN for patients treated with drugs in this class. Unlike IMiDs, proteasome inhibitors, as a class, do not appear to induce chromosomal abnormalities and therefore are not associated with increased risk for secondary malignancies. Proteasome inhibitors may therefore be safer for long-term use/maintenance therapy than other antimyeloma drugs.

**Table 2:** Selected clinical trials combining other agents with second generation proteosome inhibitors in multiple myeloma.

Compound	Phase	Combination Agents	ClinicalTrials.Gov Identifier
Carfilzomib	I, Ib and I/II	Cyclophosphamide and Dexamethasone	NCT01980589, NCT01980589 and NCT01857115
	I, I/II and I/Ib	Panobinostat	NCT01549431, NCT01496118 and NCT01301807
	I/II and Ib	Bendamustine and Dexamethasone;	NCT02002598 and NCT02095834
	I	Filanesib (An inhibitor of Akt)	NCT01989325
	III	Lenalidomide and Dexamethasone;	NCT01863550
	I	Melphalan, Bendamustine	NCT02148913
	II	Clarithromycin, Lenalidomide and Dexamethasone	NCT01559935
	I/II	Lenalidomide, Vorinostat, and Dexamethasone	NCT01297764
	I/II,	Pomalidomide and Dexamethasone	NCT01665794, NCT02185820 and NCT01464034
	I/IIb	Ibrutinib (An inhibitor of Bruton’s Tyrosine Kinase)	NCT01962792
	I/II	Selinexor (Selective Inhibitor Of Nuclear Export (SINE)) and Dexamethasone	NCT02249091
	I	Reovirus	NCT02101944
Oprozomib	Ib/II	Dexamethasone, Lenalidomide and Cyclophosphamide	NCT01881789
	I/III	Pomalidomide, and Dexamethasone	NCT01999335
	Ib/II	Melphalan and Prednisone	NCT02072863
Ixazomib (MLN9708)	II	Lenalidomide and Dexamethasone	NCT02253316
	I/II	Pomalidomide and Dexamethasone	NCT02004275
	II	Cyclophosphamide and Dexamethasone	NCT02046070
	I	Panobinostat and Dexamethasone	NCT02057640
Marizomib (NPI-0052)	I	Pomalidomide and Dexamethasone	NCT02103335

### 6.1. Peripheral Neuropathy

The PN associated with bortezomib is a sensory neuropathy affecting the hands and feet in a “stocking and glove” distribution, and is frequently painful [73]. Grade ≥ 3 PN occurs in 5%–15% of patients [34,37] but is reversible in the most cases (in the Phase III VISTA trial, 60% of instances of PN showed complete resolution within a median of 5.7 months [74]). The mechanism by which bortezomib produces PN is unknown, but may be due to aggresome formation and cytoskeletal collapse in dorsal root ganglion sensory neuron axons, alterations in mitochondrial function, or other non-proteasome mediated off-target effects [33,75,76,77,78]. It is worth noting that bortezomib may exert low-level toxic effects in other cell types besides sensory neurons, but sensory neurons are long-lived cells that are not replenished by stem cells, and damage to sensory neurons are likely to be highly salient and noticed by the patient (due to pain, *etc.*). It is possible that the boron moiety is implicated in the PN since carfilzomib (which does not contain a boron atom) is much less likely to cause PN than bortezomib (the rate of Grade 3 PN with carfilzomib is only about 1% [62]).

The incidence of PN with bortezomib is reduced by subcutaneous administration [37,38,39,79] and by less frequent dosing (e.g., weekly instead of twice per week); the hypothesis to explain this phenomenon is that IV dosing achieves peak serum levels of bortezomib which exceed the threshold to cause PN, whereas the slower pharmacokinetics of subcutaneous administration deliver an effective anti-MM dose without exceeding the neuropathy threshold.

### 6.2. Hematologic Toxicity

Hematologic adverse events appear to be a class effect associated with proteasome inhibitors; all agents tested so far are associated with thrombocytopenia, neutropenia, anemia, and lymphopenia [6,7,80,81]. Differences in the tendency of the different agents to cause hematologic toxicity remains to be established as newer agents undergo further testing in clinical trials (there is hope that second generation drugs may have lower rates of hematologic toxicity). Bortezomib, carfilzomib (best studied), and ixazomib cause transient, cyclical thrombocytopenia, with platelet counts dropping and then returning to baseline prior to the next cycle of treatment [6,82]. The exact mechanism by which bortezomib causes thrombocytopenia remains to be fully elucidated. Bortezomib does not appear to adversely affect stem cell function [83]. One hypothesis is that proteasome inhibition with bortezomib prevents the activation of NF-κB which leads to impairment of platelet budding from megakaryocytes [84].

### 6.3. Herpes Zoster Reactivation

Bortezomib has been associated with a significantly increased rate of herpes zoster reactivation [31]. In the Phase 3 study of bortezomib plus melphalan and prednisone *versus* MP alone, zoster reactivation was observed in 13% of patients in the VMP group *versus* 4% in the MP group. In the subgroup of patients in the VMP group who were receiving antiviral prophylaxis, the rate of zoster reactivation was reduced to 3% [31]. Prophylaxis with either acyclovir or valacyclovir is very effective; in a retrospective review of 125 myeloma patients who were treated with bortezomib and who also received antiviral prophylaxis at the dose of acyclovir 400 mg daily (>80% of patients), 200 mg of acyclovir (<20% of patients), 250 or 500 mg of valacyclovir, or 500 mg of famciclovir, zero instances of herpes zoster reactivation were recorded [85]. Low dose acyclovir (200 mg PO daily) has been shown to be an effective prophylactic strategy; in a retrospective analysis of 91 patients treated with bortezomib who receive low dose acyclovir, only one patient developed herpes zoster reactivation [86]. The increased susceptibility to herpes zoster reactivation in patients treated with bortezomib may be due to the effect of bortezomib treatment on the number and function of specific lymphocyte subsets [87].

### 6.4. Other Toxicities

Infusion reactions (chills, fever and dyspnea) have been observed with carfilzomib [88]. Therefore, it is recommended that dexamethasone (4 mg orally or IV) be administered prior to each dose during cycle 1 and prior to the first of the higher doses during cycle 2. Carfilzomib has been associated with pulmonary complications, renal toxicity, and cardiac events (including congestive heart failure and cardiac arrest) [62]. Chest pain and acute congestive heart failure may have been related to prehydration with normal saline. Ixazomib may cause transient rash [6]. Marizomib has been reported to cause reversible CNS toxicities (hallucinations, loss of coordination) [6]. The toxicities of the newer agents will ultimately be important in determining whether these drugs are suitable for first-line use (lower toxicities than bortezomib, on balance) or should be reserved for use in the relapsed/refractory setting (if the incidence of serious toxicities exceeds bortezomib).

## 7. Future Directions for the Use of Proteasome Inhibitors in Multiple Myeloma

### 7.1. Targeting the Immunoproteasome

In addition to the constitutive proteasome found in all cell types, a subtype immunoproteasome composed of alternative catalytic subunits is expressed abundantly in hematopoietic cells, including plasma cells and MM cells [89]. The purpose of the immunoproteasome is thought to be to produce peptide fragments optimized for MHC-1 binding and antigen presentation. Bortezomib and carfilzomib are broadly-targeted proteasome inhibitors capable of inhibiting both the constitutive and immunoproteasome subtypes; one approach under investigation to improve targeting to MM cells and reduce toxicity due to off-target drug activity in normal cells is to selectively target the immunoproteasome. Selective immunoproteasome inhibitors are under development that exploit structural differences between the two proteasome subtypes to preferentially inhibit the immunoproteasome at subnanomolar concentrations [67,90,91]. If this strategy is clinically effective, these drugs could be used instead of broadly targeted drugs (to reduce toxicities), or in combination to overcome proteasome inhibition resistance in patients with refractory disease [91,92].

### 7.2. Bone-Targeted Proteasome Inhibitors

MM causes bone resorption and lytic bone lesions via release of cytokines and growth factors that promote the growth of myeloma cells and the activation of osteoclasts. Bisphophonates are widely used in MM to present skeletal related events (pathologic fractures due to weakening of the bone and destruction by lytic lesions). Bisphosphonates bind to calcium in bone, then inhibit osteoclast activity when ‘ingested’ by osteoclasts during bone resorption. One approach to target proteasome inhibitors to bone and hence to MM is to conjugate the proteasome inhibitor to a bisphosphonate; compounds with these properties have been developed and are in the early pre-clinical stage of development [93].

### 7.3. Novel Combination Drug Therapy

Given the efficacy of proteasome inhibition in MM and the manageable side effect profile, combination therapies with many new agents are being actively tested. In addition to combinations of the new proteasome inhibitors with chemotherapy regimens with established performance in MM (including melphalan, prednisone, cyclophosphamide, *etc.*), trials are underway which involve novel IMiDs, monoclonal antibodies, histone deacetylase inhibitors, cell cycle inhibitors, aurora kinase inhibitors, other kinase inhibitors, heat-shock protein inhibitors, AKT inhibitors, mTORC1 inhibitors, and PARP inhibitors. An excellent review of novel approaches being tested in MM has been published this year by the International Myeloma Working Group [70]. Combinations with newer cytotoxic chemotherapies such as bendamustine [94], and pegylated liposomal doxorubicin [95] are also being explored. Given the manageable toxicity profile of proteasome inhibitors and the remarkable effectiveness in MM, it is likely that this class of drugs will continue to be used in the upfront and relapsed setting. Novel agents that are currently in development will likely be added on to proteasome inhibitor therapy, to restore proteasome inhibitor sensitivity.

## 8. Conclusions

MM represents a highly rationale target for proteasome inhibitors since these agents selectively target cancers that are under intrinsic endoplasmic reticular stress. Their clinical use in plasma cell diseases has led to significant improvements in outcomes for these patients. Current efforts are focused at improving efficacy through combination therapies and the clinical development of newer agents capable of overcoming resistance to bortezomib. PN remains a major problem for patients treated with bortezomib. Newer less neurotoxic toxic agents, some of which are orally active, are now available and are under evaluation in the frontline and relapsed/refractory setting.
